# Mapping 3′ transcript ends in the bank vole (*Clethrionomys glareolus*) mitochondrial genome with RNA-Seq

**DOI:** 10.1186/s12864-015-2103-2

**Published:** 2015-10-26

**Authors:** Silvia Marková, Karolína Filipi, Jeremy B. Searle, Petr Kotlík

**Affiliations:** Laboratory of Molecular Ecology, Institute of Animal Physiology and Genetics, the Czech Academy of Sciences, Rumburská 89, 27721 Liběchov, Czech Republic; Department of Genetics and Microbiology, Faculty of Science, Charles University in Prague, Viničná 5, 12844 Prague 2, Czech Republic; Department of Ecology and Evolutionary Biology, Cornell University, Ithaca, NY 14853 USA

**Keywords:** Bicistronic transcript, Mitochondrial genome, *Myodes glareolus*, Transcriptome, Polyadenylation, Stop codon

## Abstract

**Background:**

Although posttranscriptional modification of mitochondrial (mt) transcripts plays key roles in completion of the coding information and in the expression of mtDNA-encoded genes, there is little experimental evidence on the polyadenylation status and the location of mt gene poly(A) sites for non-human mammals.

**Results:**

Poly(A)-enriched RNA-Seq reads collected for two wild-caught bank voles (*Clethrionomys glareolus*) were mapped to the complete mitochondrial genome of that species. Transcript polyadenylation was detected as unmapped adenine residues at the ends of the mapped reads. Where the tRNA punctuation model applied, there was the expected polyadenylation, except for the *nad5* transcript, whose polyadenylated 3′ end is at an intergenic sequence/cytochrome *b* boundary. As in human, two pairs of bank vole genes, *nad4l*/*nad4* and *atp8/atp6*, are expressed from bicistronic transcripts. TAA stop codons of four bank vole protein-coding genes (*nad1*, *atp6, cox3* and *nad4*) are incompletely encoded in the DNA and are completed by polyadenylation. This is three genes (*nad2*, *nad3* and *cob*) less than in human. The bank vole *nad2* gene encodes a full stop codon (TAA in one vole and TAG in the other), which is followed by a 2 bp UTR and the gene conforms to the tRNA punctuation model. In contrast, the annotations of the reference mouse and some other rodent mt genomes in GenBank include complete TAG stop codons in both *nad1* and *nad2*, which overlap downstream *trnI* and *trnW*, respectively. Thus the RNA-Seq data of bank voles provides a model for stop codons of mt-encoded genes in mammals comparable to humans, but at odds with some of the interpretation based purely on genomic data in mouse and other rodents.

**Conclusions:**

This work demonstrates how RNA-Seq data were useful to recover mtDNA transcriptome data in a non-model rodent and to shed more light on mammalian mtDNA transcriptome and post-transcriptional modification. Even though gene content and organisation of mtDNA are strongly conserved among mammals, annotations that neglect the transcriptome may be prone to errors in relation to the stop codons.

## Background

Despite the growing number of animal species with completely sequenced mitochondrial (mt) genomes, surprisingly little is known about the mt transcriptome, even for mammals. Several recent papers have reviewed the mammalian mt transcriptome based on human data [1–3], while for other mammals even the basic characteristics, including the location of the transcript ends or presence of untranslated regions (UTRs) and intergenic sequences (IGSs), have not yet been described. This is surprising given that the mt genome sequence of the mouse (the first non-human mammal sequenced) has been known for over 30 years [4].

The mt genome of mammals typically consists of ~16.5 kb circular, double-stranded DNA that contains 37 genes coding for 13 protein subunits of the oxidative phosphorylation (OXPHOS) system, two ribosomal RNAs (rRNAs; rrnS and rrnL; the gene nomenclature follows that proposed by Boore [5], and adopted by e.g. Bernt [6]), and 22 transfer RNAs (tRNAs). The individual genes are unequally distributed between the heavy (H) and light (L) strands, with the majority of the genes encoded on the H strand, except for the *nad6* gene and eight of tRNAs.

Each strand of human mtDNA has its own promoter(s) and both strands are transcribed as polygenic or polycistronic precursor transcription units, which are processed to release tRNAs, rRNAs and mRNAs [1]. There are two H-strand promoter/transcription initiation sites, one located in the control region (HSP1) and one near the 5′ end of rrnS (HSP2), and one L-strand promoter located in the control region (LSP) [1]. Both HSP1 and HSP2 initiate transcription in the same direction, but the transcription from HSP2 produces a full-length transcript covering the two rRNAs, 12 mRNAs and 13 tRNAs, while the transcription from HSP1 terminates downstream of rrnL, transcribing only *trnF*, *rrnS*, *trnV* and *rrnL* [7, 8]. The existence of two distinct promoters is probably the basis for the differential regulation of synthesis of the rRNAs and H-strand encoded mRNAs, with the transcription from HSP1 being responsible for the synthesis of the majority of rRNA [7]. Similar to HSP2, the transcription initiated at LSP covers a large portion of the mt genome and includes the one mRNA (*nad6* gene) and the eight tRNAs encoded on the L strand [1].

Most of cleavage events required to release the mRNAs, tRNAs and rRNAs from these precursor transcripts can be accounted for by the ‘tRNA punctuation’ model [9]. According to the model, tRNAs are endonucleolytically excised from the precursor transcripts and the fragments that remain are processed to functional mRNA and rRNA transcripts. The 5′ and 3′ ends of the mRNA and rRNA transcripts are therefore defined by the endpoints of the intervening tRNA sequences [10]. This model ensures that adjacent mRNA and tRNA genes do not overlap or a tRNA excision would result in truncated mRNA (similar arguments apply for rRNA genes). A striking, well-demonstrated feature of human mt transcription in which it deviates from the tRNA punctuation model is that two transcription units remain as bicistronic elements containing overlapping open reading frames (ORFs) encoding *atp8* and *atp6*, and *nad4l* and *nad4*, respectively [1]. Such overlapping ORFs are present in mtDNA sequences of other mammals too, but a presence of mature mt bicistronic mRNAs in mammals other than human remains to be demonstrated.

**Fig. 1:**
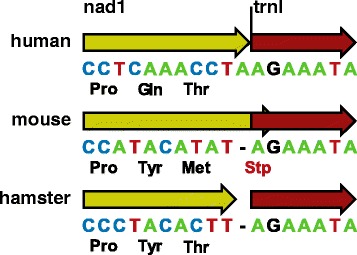
Mixed annotation of the 3′ end of *nad1* in different mammals. GenBank sequences and annotations of the *nad1* CDS (yellow) and of *trnI* (red) are shown for human (NC_012920), mouse (NC_005089) and golden hamster (NC_013276)

Due to the absence of transcriptome data, annotation of mt genomes of non-human mammals relies on homology with the human genome or is purely the result of interpretation of the mt genome sequence. For example, there are two instances of an overlapping TAG stop codon (in *nad1* and *nad2*) and downstream tRNA in the NCBI Reference Sequence of the house mouse (*Mus musculus*) mitochondrion (GenBank:NC_005089.1; as of February 2015) in an annotation apparently inferred solely from the mt genome sequence, while in human the stop codons of these genes are TAA codons incompletely encoded in DNA with the missing adenine residues added via mRNA polyadenylation (Fig. 1) [1]. Among other rodent mt genomes, some match the mouse annotation, including the Norway rat *Rattus norvegicus* NCBI Reference Sequence (GenBank:AC_000022.2) and the sequence of the sibling vole *Microtus rossiaemeridionalis* (GenBank:DQ015676.1), while others conform to the human model, including the golden hamster *Mesocricetus auratus* NCBI Reference Sequence (GenBank:NC_013276.1) (Fig. 1) and the Korean red-backed vole *Eothenomys regulus* (GenBank:JN629046.1). The eastern European house mouse *Mus musculus musculus* (GenBank:KF781649.1) annotation also follows human rather than the house mouse (strictly the western European house mouse *Mus musculus domesticus*) annotation.

**Fig. 2:**
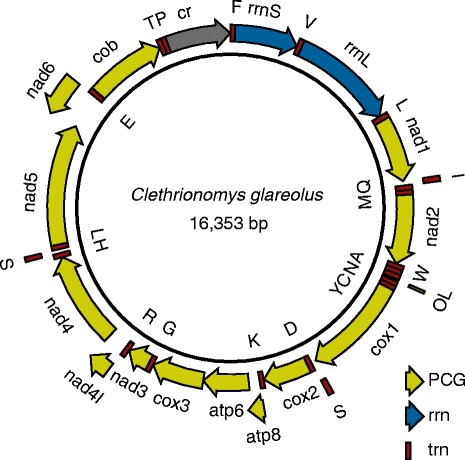
The mitochondrial genome of the bank vole. Arrows pointing clockwise indicate rRNA and protein-coding genes (PCGs) encoded by the heavy strand and counter-clockwise arrow PCG encoded by the light strand

In this study, we characterise the mt transcriptome of a cricetid rodent, the Eurasian bank vole *Clethrionomys glarolus*. As for other non-human mammals, there is no experimental evidence of the ends of the bank vole mRNA molecules and their polyadenylation status. Recently, we sequenced and annotated the mt genome of the bank vole based on Sanger sequencing (Fig. 2) [11, 12]. Here, we utilize Illumina RNA-Seq data collected for two wild-caught individuals to describe the mt transcriptome of the bank vole. Although RNA-Seq data are primarily employed to gather information about nuclear-encoded transcripts, they typically contain a high proportion of reads matching the mt transcripts, potentially extremely useful in mt transcriptome characterization [13, 14]. We mapped the bank vole RNA-Seq reads to the sequence of the mt genome and characterise the 3′ends of the mRNAs from the bank vole mt genome. On the basis of this information, we assess the polyadenylation status of mRNAs, the codon usage to terminate the translation, and the presence of UTRs and IGSs in the bank vole mtDNA.

## Results

**Fig. 3:**
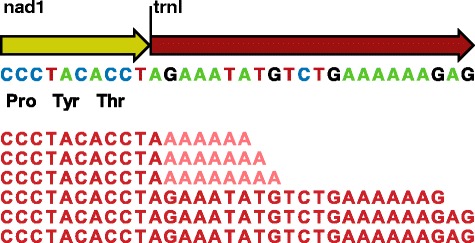
Location of the transcript 3′ ends by RNA-Seq. The *nad1* gene is shown here as an example of the way in which reads with poly(A) tails can be mapped with unaligned ends (colour-coding as in Fig. 1). The transcript polyadenylation sites are identified as stretches of adenine residue that do not map back to the genome and lie at the ends of mapped fractions of the reads (the top three reads). The three reads at the bottom of the mapping match the genomic sequences along their entire length and most likely derive from unprocessed polycistronic transcript

Our mapping strategy allowed the identification of transcript polyadenylation as stretches of three or more adenine residue that do not map back to the genome and lie at the ends of mapped fractions of the reads mapping to the 3′ ends of protein-coding genes (PCGs; Fig. 3) [15]. Locating the poly(A) sites made it possible to determine where the stop codon of PCGs is completed by polyadenylation, including for the genes where a triplet TAA, TAG or TGT overlaps the first one or two nucleotides of the downstream gene. Knowing the location of the poly(A) sites also allowed the identification of the 3′ ends of the transcripts and the search for the presence of UTRs and of non-coding IGSs.

### Mapping statistics

**Fig. 4:**
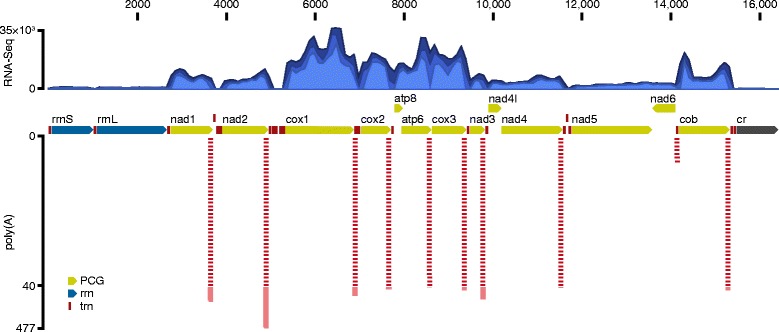
Read mapping to the mitochondrial genome. Coverage of the bank vole mtDNA by the RNA-Seq reads (top panel) and the distribution of the reads matching the poly(A) 3′ transcript ends (combined evidence from the two voles; bottom panel). Annotation arrows pointing right indicate RNA and protein-coding genes (PCGs) encoded by the heavy strand and left arrows PCG encoded by the light strand. In the top panel, the minimum, mean and maximum coverage values are shown in different shades of blue as calculated using a window size of 100 bp. In the bottom panel, the total number of individual reads that align to the transcript ends are shown, with those between 1 and 40 displayed by a ladder (linear scale), with any additional reads between 41 and 477 displayed as a solid bar (linear scale)

A total of 50,610,204 and 50,206,006 paired reads were generated for the voles 1634 and 1815 (P. Kotlík specimen database), respectively. Of these 923,507 and 1,015,279 reads (i.e. 2 % of total reads) were mapped to the 16,353-bp-long mt genome (Fig. 2), with the average coverage of 5696× and 6248×, respectively. Of total mapped reads, 892,875 and 992,592 reads (97 and 98 %) mapped to the 13 PCGs (11,390 bp in total; Fig. 4) in the two voles, respectively. The average coverage of PCGs was 7928× and 8454×, respectively, with the coverage of 100× or higher over 99 % of the nucleotides. The average coverage of each position in the last 100 bp of the PCGs, that is in the region critical for poly(A) detection, ranged from 2580× to 5010× in vole 1634 and from 3035× to 10,697× in vole 1815.

### Transcript polyadenylation sites

**Fig. 5:**
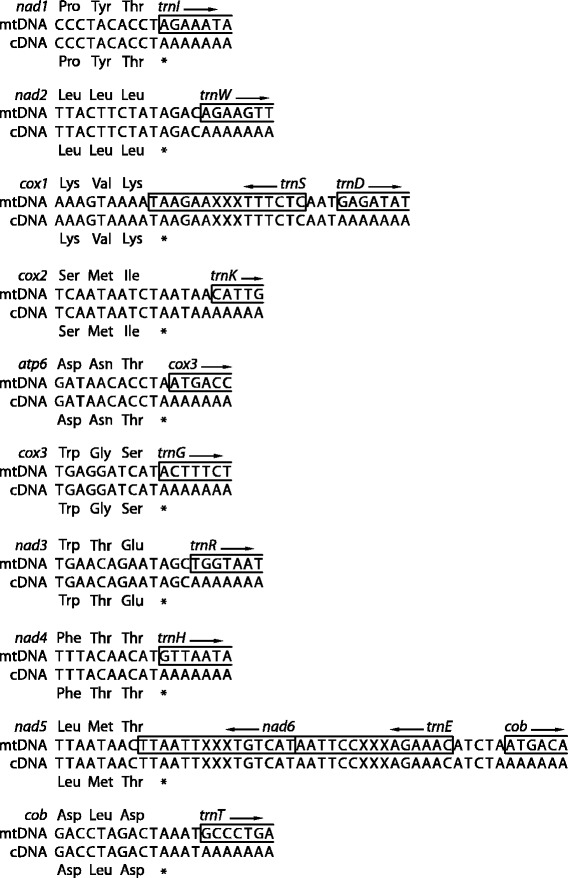
Alignment of RNA-Seq reads with poly(A) ends to mitochondrial genes. The last three codons and the stop codon are shown for each protein-coding gene (PGS) encoded on the heavy strand except for the upstream genes from both bicistronic transcripts (*nad4l* and *atp8*). An example poly(A) end-read (cDNA sequence) is shown aligned to the genomic (mtDNA) sequence. The sequence of the gene located immediately downstream of each PGC is shown over a length corresponding to the unaligned poly(A) end of the read. Only the first and last six nucleotides are shown in antisense sequences of the genes contained within the 3′ untranslated regions of *cox1* (*trnS*) and *nad5* (*trnE* and *nad6*), with their middle sections represented by ‘XXX’

Clear signatures of 3′ end transcript polyadenylation were found for 10 of the 12 PCGs encoded on the H-strand (Fig. 4). The poly(A) stretches started from 0 bp (*nad1*, *atp6*, *cox3*, *nad4*) to 595 bp (*nad5*) downstream of the last DNA-encoded nucleotide of the stop codon (Fig. 5). A minimum of 40 reads with three or more non-template adenine nucleotides at the 3′ end mapped to the putative transcript end of each of these genes (Fig. 4). The only exception was the *nad5* transcript, to which only seven poly(A) end-reads were mapped (Fig. 4). For the other genes, the number of poly(A) end-reads was 194 (*nad1*), 477 (*nad2*), 131 (*cox1*), 64 (*cox2*), 44 (*atp6*), 75 (*cox3*), 170 (*nad3*), 45 (*nad4*), and 73 (*cob*). These results allowed unambiguous assignment of the location of the poly(A) sites and thus mature transcript ends for each H-strand PCG (Fig. 5).

### Posttranscriptional stop-codon completion

Rather than a complete stop codon in the DNA, three of the PCGs (*nad1*, *cox3* and *nad4*) have a solitary T residue before the 5′end of a downstream tRNA, and in the case of the *atp6* gene, a TA before the 5′end of the downstream adjacent *cox3* gene (Fig. 5). In all these genes, the poly(A) site was located immediately following the last DNA-encoded nucleotide of the stop codon, i.e. T in the case of *nad1* (Fig. 3), *cox3* and *nad4*, and A in the case of *atp6*. Therefore, in these four genes a TAA stop codon was created by post-transcriptional polyadenylation that changed T or TA residues to the complete TAA stop codon (Fig. 5). A TAA stop codon thus terminates the translation of all mRNAs in the bank vole mtDNA. The exceptions are *nad2* and *nad3* terminating with TAA in the vole 1815, but with TAG in the vole 1634.

### Bicistronic transcripts with overlapping reading frames

No poly(A) end-reads mapped to the 3′ ends of *atp8, nad4l* and *nad6*. The reading frames of the two H-strand genes (*atp8* and *nad4l*) overlap the reading frames of the respective downstream genes in a tail-to-head manner by 43 bp (*atp8*/*atp6*) and 7 bp (*nad4l*/*nad4*) (Fig. 2). The reading frame overlap and the absence of a poly(A) tail from the more upstream gene in each pair (*atp8, nad4l*) most likely mean that in each case the two genes are expressed from the same, bicistronic transcript (*atp8*/*atp6* and *nad4l*/*nad4*). This explains the absence of poly(A) tails downstream of the ORF of the more upstream gene in each case (*atp8* and *nad4l*; Fig. 4). It also explains why *atp8* and *nad4l* show no drop in coverage observed for the tRNA genes (Fig. 4), which is due to the library enrichment for poly(A)-tailed RNA.

### Exception to the tRNA punctuation model

There is also no intervening tRNA sequence between the *atp6* and *cox3* genes (Figs. 2 and 5). The reading frames of neighbouring PCGs in the bank vole mt genome are interrupted with tRNA genes, with the exception of the two pairs of overlapping genes (*atp8/atp6*, *nad4l/nad4*; see above) and of the *atp6* and *cox3* genes, which are attached to each other in a tail-to-head manner (Fig. 2). However, unlike the more upstream genes associated with the two bicistronic transcripts (*atp8* and *nad4l*), both *atp6* and *cox3* transcripts showed clear evidence of polyadenylation (Fig. 4), which completed the TAA stop codon of both these genes (Fig. 5). Therefore, the bank vole *cox3* gene is expressed from a different mature mRNA than the *atp8/atp6* transcript. These transcripts thus represent an apparent exception to the tRNA punctuation model in that another mechanism needs to be postulated to account for the cleavage site between *atp6* and *cox3*.

### Untranslated and intergenic regions

Mature transcripts of six PCGs contained a UTR at the 3′ end, defined as the region between the stop codon and the poly(A) site. Most 3′ UTRs were a single bp (C in *nad3*) or a few bp (AC in *nad2*, AT in *cob* and TAA in *cox2*) long. However, two monocistronic mRNA transcripts contained substantially longer 3′ UTRs (Fig. 5). First, the *cox1* transcript contained a 69 bp long 3′UTR corresponding to the full antisense sequence of *trnS* followed by 3 bp of IGS sequence (AAT; Fig. 5; see below). Second, the 595 bp long *nad5* 3′UTR contained full antisense sequences of *nad6* and *trnE* (*nad5* and *nad6* overlap tail-to-tail by 4 bp), and a 5 bp IGS (ATCTA; Figs. 4 and 5).

**Fig. 6:**
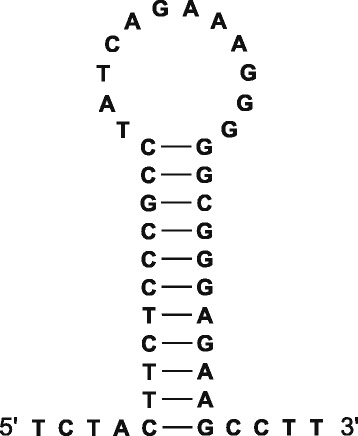
Predicted stem-loop structure of the light-strand origin of replication (O_L_). The free energy of the structure was −21.6 kcal/mol, as calculated with the CLC Genomics Workbench using the algorithm in [24]

The largest IGS in the bank vole mt genome, the control region (displacement loop) flanked by *trnP* and *trnF*, was 946 bp long in both individuals analysed. The second major IGS, 31 bp long in both mitogenomes, was located within a cluster of five tRNA genes (*trnW*, *trnA*, *trnN*, *trnC* and *trnY*), specifically between *trnN* and *trnC* (Fig. 2). This region has the capacity to fold into a stable stem-loop secondary structure (Fig. 6) and most likely represents the L-strand origin of replication (O_L_). Additionally, there were 12 short IGSs. These IGSs were from 1 to 5 bp long, totalling up to 26 bp and covering 0.16 % of the bank vole mitogenome, and were identified mostly between PCGs and tRNA genes or between different tRNA genes (Table 1).

**Table 1:** Intergenic sequences in the bank vole mitochondrial genome

Flanking genes	Position	Length (bp)	Type of sequence
*trnF/rrnS*	67–68	2	Non-coding
*nad2/trnW*	4931–4932	2	Non-coding
*trnW/trnA*	5000	1	Non-coding
*trnA/trnN*	5070–5071	2	Non-coding
*trnN/trnC*	5142–5172	31	Origin of light-strand replication, O_L_
*trnY/cox1*	5308	1	Non-coding
*trnS2/trnD*	6920–6922	3	Non-coding
*trnD/cox2*	6991	1	Non-coding
*cox2/trnK*	7676–7678	3	Non-coding
*nad3/trnR*	9785	1	Non-coding
*trnR/nad4l*	9854–9856	3	Non-coding
*trnE/cob*	14,123–14,127	5	Non-coding
*cob/trnT*	15,271–15,272	2	Non-coding
*trnP/trnF*	15,408–16,353	946	Control region

## Discussion

Although posttranscriptional modification of mt transcripts plays key roles in complementation of the coding information and in the expression of mtDNA-encoded genes, there is little experimental evidence on the polyadenylation status and the location of poly(A) sites in mtDNA for mammals other than humans [16, 17]. The present study starts to fill this gap by using RNA-Seq reads derived from poly(A)-enriched bank vole RNA to characterise the 3′ ends of the mt transcripts in this species.

The general characteristics of the bank vole mt transcriptome match the human model. All PCGs except one (*nad6*) are transcribed from the H-strand. The H-strand transcriptome consist of ten monocistronic and two bicistronic mRNAs. All bank vole transcripts punctuated by tRNA genes at their 3′ ends are polyadenylated (Fig. 4), as found for human mtDNA [18]. Our data demonstrate that the polyadenylation of bank vole mt transcripts occurred precisely at the sites predicted by the tRNA punctuation model, except for the *nad5* transcript, whose 3′ end is at an IGS/*cob* boundary (Fig. 5). This deviation from the tRNA punctuation model matches previous findings in humans [1]. The two mature bicistronic transcripts correspond to the mRNA for *atp8/atp6* subunits and the mRNA for *nad4l*/*nad4* subunits, with ORFs overlapping by 43 and 7 bp, respectively. The expression of these genes from bicistronic units has been demonstrated in humans [1], and although the ORFs similarly overlap in mtDNA of other mammals [4, 19], only for the bank vole have we provided experimental evidence in support of the existence of mature bicistronic transcripts. This shows that this feature of mtDNA transcription is shared by rodents and primates.

Our RNA-Seq data provided no evidence for the location of the 3′ end of the mRNA for *nad6*. This is the only PGC encoded on the L-strand [11]. The ORFs of bank vole *nad5* and *nad6* overlap tail-to-tail for 4 bp and the antisense sequence of *nad6* is fully contained in the 3′ UTR of *nad5* (Fig. 5). However, the bank vole *nad6* ORF is not punctuated by a downstream tRNA and its mRNA does not appear to be stably polyadenylated near the 3′ end of the ORF, consistent with human [1, 18]. The location of the end of the bank vole *nad6* transcript therefore remains unknown as does its polyadenylation status. Thus, while our approach allowed accurate positioning of the 3′ ends of major transcripts for all H-strand PCGs (Fig. 4), RNA extracted from enriched mitochondria and/or RT-PCR or deep sequencing strategies specifically targeting transcript ends will be required to describe the ends of the L-strand encoded transcript(s) as well as of rrnS and rrnL transcripts, and to test for possible additional minor poly(A) sites [2, 20].

Four of the bank vole PCGs (*nad1*, *atp6, cox3* and *nad4*) have TAA stop codons that are incompletely encoded in the DNA and are completed posttranscriptionally by polyadenylation, which is demonstrated by the presence of a poly(A) site immediately downstream of the last DNA-encoded stop codon nucleotide (T or A; Fig. 5). This finding is in concordance with Slomovic et al. [18] who found in human cells that polyadenylation is a key step in completion of stop codons only partly encoded by mtDNA. Stop codons of seven PCGs in human mtDNA (including *nad2*, *nad3* and *cob*) require completion by polyadenylation [1]. Therefore, while the mechanism is shared by the two species, there is heterogeneity in the role of polyadenylation in completion of the coding information of the individual genes between human and the bank vole as some of the genes with incomplete stop codons in human encode full stop codons in the bank vole. For example, while the stop codon of *nad1* is only partly encoded in the DNA (TA and T, respectively) and is posttranscriptionally completed to TAA in both species (Fig. 1) [1], *nad2* encodes an incomplete stop codon (T) in human, but a full stop codon in the bank vole (TAA in one vole and TAG in the other), where it is further followed by 2 bp of UTR sequence (AC) (Fig. 5). The poly(A) site immediately follows this short 3′UTR (Fig. 5), which demonstrates that the bank vole *nad2* mRNA also fully conforms to the tRNA punctuation model, but that in this species polyadenylation is not required to complete the stop codon.

In contrast to the evidence from human [1] and bank vole, annotations of various rodent mt genome sequences in GenBank (including the reference mouse mt genome) include complete TAG stop codons in the CDS of both *nad1* and *nad2* that overlap the downstream *trnI* and *trnW* by 2 bp (AG) in each case (Fig. 1). Such CDS/tRNA overlap would imply a deviation from the tRNA punctuation model [9] and an alternative RNA processing mechanism would need to be postulated. The discordance in *nad1* and *nad2* annotation among different species reflects insufficient transcriptome data. We believe that unambiguous determination of stop codons is, in some cases, not possible based solely on genomic sequence.

## Conclusions

The present study is a demonstration of how deep RNA-Seq from total RNA was useful to recover mtDNA transcriptome data in a non-model rodent species. This approach has previously been applied to study mt gene expression of an insect, including polyadenylation and polycistronic transcripts [14]. We show that RNA-Seq data are helpful in evaluation of gene boundaries, polyadenylation and post-transcriptional modification in mammalian mtDNA. Even though the structure, genetic content and organization of mtDNA are strongly conserved among mammals and human mtDNA is considered a paradigm for the whole class [3], it is imperative that sequencing of new mt genomes incorporate evidence from the transcriptome as often as possible, as annotations based on genomic data only are liable to errors. Fortunately, typical mammalian whole transcriptome RNA-Seq data contain a high proportion of reads matching the mt transcripts that remain largely unexplored in most gene expression studies, but which can be extremely useful in mt transcriptome characterization. This includes RNA-Seq data from published studies that are available in read archives. As Smith [21] points out, ‘the bulk of the organelle-derived RNA-Seq reads in public databases are waiting to be analysed’. Therefore, for some species (such as the mouse), it should be possible to rectify the situation even with the data that are now available.

## Methods

### Samples and RNA extraction

RNA-Seq data were collected for two adult bank voles from England, a male from near Diptford in Devon (vole no. 1634; P. Kotlík specimen database) and a female from near Cirencester in Gloucestershire (vole no. 1815). The individuals were trapped and taken under the appropriate Natural England (general) licence for wildlife management, strictly following the guidelines therein. The bank vole is a widespread Eurasian rodent species of the family Cricetidae, which is taxonomically placed within the same superfamily Muroidea as the house mouse (which is in the family Muridae). Total RNA was extracted with RNeasy Mini Kit (Qiagen, Valencia, CA, USA) from spleen samples stored in RNAlater, followed by DNase treatment using TURBO DNA-free Kit (Ambion, Austin, TX, USA) and an additional clean-up step using the RNeasy Mini Kit.

### Illumina sequencing

Library preparation and sequencing were performed with standard Illumina (San Diego, CA, USA) protocols on the Illumina HiSeq 2000 as described previously [12, 22, 23]. Briefly, mRNA molecules with stretches of poly(A) residues at the 3′ end were separated from RNAs that lack a poly(A) tail. After the poly(A) enrichment and fragmentation, the RNA was size-selected to 250–400 bp, reverse transcribed into cDNA, end-repaired and PCR-enriched. The resulting libraries were sequenced using the 100-bp paired-end module.

### Mapping reads to the genome

The Illumina reads obtained for each vole were mapped to the annotated reference sequence of the bank vole mt genome (GenBank:KF918859) [11]. Read mapping was performed using the CLC Genomics Workbench, version 6.0.1 (CLC bio A/S, Aarhus, Denmark), with a minimum length fraction of 0.9 and minimum similarity fraction of 0.95. This means that only those reads were considered where at least 90 % of the read matched the reference sequence with at least 95 % identity. Such a setting ensured the specificity of the mapping, but allowed for the reads with poly(A) tails to be mapped with unaligned ends. Reads that mapped with unaligned three or more non-template adenine nucleotides at the 3′ end were considered indicative of poly(A) sites, a criterion similar to that adopted by previous studies [2, 14]. To ascertain the location of the ends of the tRNA genes, the cloverleaf secondary structure of each tRNA was predicted using MITOS [6]. The secondary structure of the putative O_L_ was predicted with the CLC Genomics Workbench.

## References

[1] Temperley RJ, Wydro M, Lightowlers RN, Chrzanowska-Lightowlers ZM (2010). Human mitochondrial mRNAs-like members of all families, similar but different. Biochim Biophys Acta.

[2] Mercer TR, Neph S, Dinger ME, Crawford J, Smith MA, Shearwood AM (2011). The human mitochondrial transcriptome. Cell.

[3] Peralta S, Wang X, Moraes CT. Mitochondrial transcription: Lessons from mouse models. Biochim Biophys Acta. 2012;1819:961–9. 10.1016/j.bbagrm.2011.11.001 PMC3408808 22120174

[4] Bibb MJ, Van Etten RA, Wright CT, Walberg MW, Clayton DA (1981). Sequence and gene organization of mouse mitochondrial DNA. Cell.

[5] Boore JL (2006). Requirements and standards for organelle genome databases. OMICS.

[6] Bernt M, Donath A, Juhling F, Externbrink F, Florentz C, Fritzsch G (2013). MITOS: improved de novo metazoan mitochondrial genome annotation. Mol Phylogenet Evol.

[7] Montoya J, Gaines GL, Attardi G (1983). The pattern of transcription of the human mitochondrial rRNA genes reveals two overlapping transcription units. Cell.

[8] Martin M, Cho J, Cesare AJ, Griffith JD, Attardi G (2005). Termination factor-mediated DNA loop between termination and initiation sites drives mitochondrial rRNA synthesis. Cell.

[9] Ojala D, Montoya J, Attardi G (1981). tRNA punctuation model of RNA processing in human mitochondria. Nature.

[10] Rossmanith W, Tullo A, Potuschak T, Karwan R, Sbisa E (1995). Human mitochondrial tRNA processing. J Biol Chem.

[11] Bendová K, Marková S, Searle JB, Kotlík P. The complete mitochondrial genome of the bank vole *Clethrionomys glareolus* (Rodentia: Arvicolinae). Mitochondrial DNA. 2014:doi:10.3109/19401736.2013.873927. 10.3109/19401736.2013.873927 24438307

[12] Filipi K, Marková S, Searle JB, Kotlík P (2015). Mitogenomic phylogenetics of the bank vole *Clethrionomys glareolus*, a model system for studying end-glacial colonization of Europe. Mol Phylogenet Evol.

[13] Torres TT, Dolezal M, Schlotterer C, Ottenwalder B (2009). Expression profiling of *Drosophila* mitochondrial genes via deep mRNA sequencing. Nucleic Acids Res.

[14] Wang HL, Yang J, Boykin LM, Zhao QY, Li Q, Wang XW (2013). The characteristics and expression profiles of the mitochondrial genome for the Mediterranean species of the *Bemisia tabaci* complex. BMC Genomics.

[15] Waern K, Snyder M (2013). Extensive transcript diversity and novel upstream open reading frame regulation in yeast. Genes Genomes Genetics.

[16] Taanman JW (1999). The mitochondrial genome: structure, transcription, translation and replication. Biochim Biophys Acta.

[17] Nagaike T, Suzuki T, Ueda T (2008). Polyadenylation in mammalian mitochondria: insights from recent studies. Biochim Biophys Acta.

[18] Slomovic S, Laufer D, Geiger D, Schuster G (2005). Polyadenylation and degradation of human mitochondrial RNA: the prokaryotic past leaves its mark. Mol Cell Biol.

[19] Gadaleta G, Pepe G, De Candia G, Quagliariello C, Sbisa E, Saccone C (1989). The complete nucleotide sequence of the *Rattus norvegicus* mitochondrial genome: cryptic signals revealed by comparative analysis between vertebrates. J Mol Evol.

[20] Stewart JB, Beckenbach AT (2009). Characterization of mature mitochondrial transcripts in *Drosophila*, and the implications for the tRNA punctuation model in arthropods. Gene.

[21] Smith DR (2013). RNA-Seq data: a goldmine for organelle research. Brief Funct Genomics.

[22] Kotlík P, Marková S, Vojtek L, Stratil A, Šlechta V, Hyršl P, et al. Adaptive phylogeography: functional divergence between haemoglobins derived from different glacial refugia in the bank vole. Proc R Soc B Biol Sci. 2014;281:20140021 10.1098/rspb.2014.0021 PMC4046400 24827438

[23] Marková S, Searle JB, Kotlík P (2014). Relaxed functional constraints on triplicate alpha-globin gene in the bank vole suggest a different evolutionary history from other rodents. Heredity.

[24] Zuker M, Waterman MS (1989). The use of dynamic programming algorithms in RNA secondary structure prediction. Mathematical methods for DNA Sequences.

